
JOURNAL INFORMATION
==============================
NLM Title Abbreviation: Innov Surg Sci
Journal ID: Innov Surg Sci
NLM Journal ID: 101708165
Journal ID: iss

Innovative Surgical Sciences

EISSN: 2364-7485
Publisher: De Gruyter

ARTICLE INFORMATION
==============================
PMCID: PMC13068488
DOI: 10.1515/iss-2026-2005
Article ID: iss-2026-2005
Article version: 1
Subjects: Article

5. Vascular Surgery

Electronic publication date: 2026 Apr 10
Publication date: 2026 Apr
Volume: 11
Issue: Suppl1
First page: 386
Last page: 395
Copyright: © 2026 by Walter de Gruyter Berlin/Boston
Copyright year: 2026
Copyright holder: De Gruyter
License: This work is licensed under the Creative Commons Attribution 4.0 International License.
License URL: https://creativecommons.org/licenses/by/4.0/

Page count: 10

==============================

DE GRUYTER BRILL

**Perioperative Morbidity and Mortality of Carotid Endarterectomy – 10-Year Data**
ID: E-DCK-206

**Gefäßchirurgie / Vascular Surgery**
**Gefäßchirurgie im Spannungsfeld der Krankenhausplanung / Vascular Surgery in the Context of Hospital Resource Planning**

Peter Konstantiniuk[1], Simon Thomas[1], Gregor Siegl[1], Tina Cohnert[1]

[1]Medical University of Graz, Division of Vascular Surgery, Department of Surgery, Graz, Österreich

**Background:**
The European Society of Vascular Surgery (ESVS) has published quality standards in its guidelines for Carotid Endarterectomy. The perioperative mortality and stroke rate should be below 3% for asymptomatic and below 6% for symptomatic patients. The objective of this study was to verify whether these thresholds could be maintained. Furthermore, the rate and the underlying reasons for reoperations were to be determined.

**Material and methods:**
Included were all patients who underwent surgery at the University Hospital Graz within the period from January 1st , 2014, to December 31st, 2023. A retrospective data collection was performed. To execute this analysis, all CT and MRI data, reoperations, and deaths within 30 days from the KAGES hospitals across the entire region of Styria were collected and evaluated with the help of the IT department.

**Results:**
A total of 2500 patients were evaluated, with an average age of 71.3 years. Gender distribution showed 67.6% (1689/2500) men and 32.4% (811/2500) women; 76.6% (1916/2500) were asymptomatic, while 23.4% (584/2500) were symptomatic. Sinus rhythm was present in 92.0% (2301/2500), with (intermittent) Atrial Fibrillation/Flutter (VHFA) in 7.9% (197/2500). Postoperatively, 2.3% (45/1871) of asymptomatic patients and 2.6% (15/569) of symptomatic patients required at least one reoperation. The causes were: 76.7% (46/60) bleeding, 21.7% (13/60) incorrect revascularization, and 1.7% (1/60) deep abscess. A total of 14 deaths occurred within 30 days: 0.5% (10/1906) for asymptomatic patients and 0.7% (4/580) for symptomatic patients. The stroke rate was 1.7% (32/1884) for asymptomatic patients and 4.8% (28/556) for symptomatic patients. The combined endpoint (stroke and death) occurred in 2.9% (73/2500) of patients. The risk for symptomatic patients was 5.5% (32/552), significantly higher than for asymptomatic patients at 2.1% (41/1875).

**Conclusion:**
The analysis confirms that the ESVS standards were achieved at the University Hospital Graz. There are ongoing discussions within the ESVS about whether the quality thresholds for carotid surgery should be lowered. Our data advocate for maintaining the established standards.

Open Access. © 2026 The Author(s), published by De Gruyter. This work is licensed under the Creative Commons Attribution 4.0 International License.

DE GRUYTER BRILL

### Type and Frequency of Follow-up Procedures after Endovascular Repair Infrarenal Aortic Aneurysms

ID: E-DCK-211

**Gefäßchirurgie / Vascular Surgery**
**Gefäßchirurgie im Spannungsfeld der Krankenhausplanung / Vascular Surgery in the Context of Hospital Resource Planning**

Peter Konstantiniuk[1], Noemi Jung[1], Gregor Siegl[1], Tina Cohnert[1]

[1]Medical University of Graz, Division of Vascular Surgery, Department of Surgery, Graz, Österreich

**Background:**
Endovascular repair of an infrarenal aortic aneurysm is associated with significantly lower perioperative morbidity and mortality compared to open surgery. Conversely, the rate of postoperative corrections (interventional or open surgical) is significantly higher. The aim of the present study is to demonstrate the frequency and type of postoperative corrections after stent graft implantation.

**Material and methods:**
All endovascularly treated abdominal aortic aneurysms (AAA) at the vascular surgery department of our tertiary university hospital between 2014 and 2023 were retrospectively analyzed. Exclusively infrarenal aortic aneurysms were included; suprarenal, juxtarenal, and pararenal aortic aneurysms were excluded, as were correction stent grafts, PAUs, monoiliac stent grafts, and ruptured abdominal aortic aneurysms.

**Results:**
In the specified period, 328 patients were treated endovascularly for an abdominal aortic aneurysm. In accordance with the exclusion criteria, 49 patients were excluded, and 279 cases were statistically evaluated. The mean age was 74.3 years (range 34–93), and the majority, 251 patients (90.0%), were male, and 28 patients (10.0%) were female.
Stentgraft saving follow-up procedures:

| Number of Interventional Correction Procedures | Number of Patients | Percentage |
|---|---|---|
| 0 | 209 | 76.0% |
| 1 | 45 | 16.4% |
| 2 | 12 | 4.4% |
| 3 | 6 | 2.2% |
| 4 | 2 | 0.7% |
| 5 | 1 | 0.4% |

The most common postoperative procedures were transarterial embolizations of endoleaks with 24 interventions, stent graft extensions occurred in 16 cases, and thrombectomy of a stent graft limb in 12 patients. In addition to the stent graft preserving procedures, conversion to open surgical repair occurred in 5 cases.

**Conclusion:**
When choosing the correction procedure for an infrarenal aortic aneurysm, the probability of follow-up procedures should be included in the considerations, as the follow-up monitoring of aortic stent grafts for the detection or exclusion of endoleaks requires frequent computed tomography scans with associated radiation exposure and potential nephrotoxicity.

DE GRUYTER BRILL

**Open vs. Endovascular Repair of Abdominal Aortic Aneurysms: 13 y of experience at a Level 2 hospital**
ID: E-DCK-217

**Gefäßchirurgie / Vascular Surgery**
**Gefäßchirurgie im Spannungsfeld der Krankenhausplanung / Vascular Surgery in the Context of Hospital Resource Planning**

Steffen Güth[1], Michael Trassl[1], Wolfgang Hoffmann[2], Valerie Nikolov[3], Franck Billmann[3 4]

[1]Klinikum Heidenheim, Viszeral-, Thorax- und Gefäßchirurgie, Heidenheim, Deutschland, [2]Klinikum Heidenheim, Klinik für Viszeral-, Thorax- und Gefäßchirurgie, 89522 Heidenheim an der Brenz, Deutschland, [3]Klinikum Heidenheim, Viszeral-, Thorax- und Gefäßchirurgie, 89522 Heidenheim an der Brenz, Deutschland, [4]Universität Heidelberg, Im Neuenheimer Feld 672, 69120 Heidelberg, Deutschland

**Background:**
Abdominal aortic aneurysms (AAA) are a substantial burden on health care in more developed countries, occurring mostly among men older than 65 years of age. Although underestimated, rupture of these aneurysms (rAAA) causes about 8000 deaths per year in the UK and roughly 2 000 per year in the Germany. In general, the risk of rupture increases as the diameter of the aneurysm enlarges. The overall mortality rate for patients with ruptured abdominal aortic aneurysms is between 65% and 85%, and about half of deaths attributed to rupture occur before the patient reaches the surgical room. The treatment of intact abdominal aortic aneurysm is essentially prophylactic and aimed at prevention of fatal rupture. By contrast with the progress in elective repair, no improvement in operative mortality of ruptured aneurysms has been reported during the past decades, remaining as high as 30–70%. It is possible to differentiate between the open surgical approach and the endovascular approach. In this series, we present the experience of our level 2 hospital in different types of surgical treatments.

**Material and methods:**
Prospectively collected data on all patients who underwent AAA repair (asymptomatic and ruptured) between January 2012 and January 2025 were assessed retrospectively. Demographic, clinical and perioperative data were analyzed. A descriptive analysis and assessment of the following endpoints was carried out: 30 days morbidity (including endoleak, spinal ischemia, intestinal ischemia, infectious complications), perioperative mortality, procedure duration, length of stay, procedure costs.

**Results:**
A total of 212 patients underwent open surgical or endovascular treatment for aAAA and/or rAAA. 162 patients underwent endovascular treatment (76.42%). Open vascular surgery was performed in 50 patients (23,58%). Overall mortality was 3.8% (all of these patients in the open rAAA repair group). Procedure duration was around 2 hours in the endovascular and 4 hours in the open group. Length of stay was respectively 4 days and 9 days.

**Conclusion:**

Indication for surgical treatment is deduced from the estimated risk of rupture, the estimated risk of the surgical procedure, and the estimated life expectancy of the patient. Endovascular treatment of AAA is safe and effective. As far as emergency repair for ruptured abdominal aortic aneurysms is concerned, mortality depends on the haemodynamic status of the patient at the time of surgery and the type of procedure used (endovascular vs open). In addition to Level 3 hospitals, Level 2 hospitals may also provide high-quality treatment for AAA.

DE GRUYTER BRILL

**Advanced analgetic treatment – what the vascular surgeon must know based on a specific "SOP"**
ID: E-DCK-244

**Gefäßchirurgie / Vascular Surgery**
**Gefäßchirurgie im Spannungsfeld der Krankenhausplanung / Vascular Surgery in the Context of Hospital Resource Planning**

Michael Brinkers[1], Giselher Pfau[1], Christof Strang[1], Uwe Lodes[2], Zuhir Halloul[3], Frank Meyer[4], Udo Barth[3]

[1]Otto-von-Guericke-Universität mit Universitätsklinikum, Klinik für Anästhesiologie und Intensivtherapie, Schmerzambulanz, Magdeburg, Deutschland, [2]AMEOS Klinikum Schönebeck, Klinik für Anästhesiologie und Intensivtherapie, Schönebeck, Deutschland, [3]Otto-von-Guericke-Universität mit Universitätsklinikum, Klinik für Allgemein-, Viszeral-, Gefäß- und Transplantationschirurgie, Arbeitsbereich Gefäßchirurgie, Magdeburg, Deutschland, [4]Otto-von-Guericke-Universität mit Universitätsklinikum, Klinik für Allgemein-, Viszeral-, Gefäß- und Transplantationschirurgie, Magdeburg, Deutschland

**Background:**
Years ago, the interdisciplinary working group of the University of Magdeburg published a “Standard operating procedure” (SOP) for postoperative pain therapy in general surgery.
*Aim*: It was necessary to adapt the SOP “Postoperative pain therapy” to vascular surgery.

**Material and methods:**
Narrative short review based on the developed principles and policies of the “Standard Operating Procedure“ (SOP) - “Pain Therapy in Vascular Surgery“ (will be provided as part of the presentation)

**Results:**
(key points): While most vascular changes due to disease (e. g., rheumatoid arthritis, vasculitis) are present in the per mille range in the population, some of the changes due to civilization (ulcus cruris, chronic wound) and increasing age based on the higher life expectancy are present in the double-digit percentage range and continue to increase in incidence.
They are initially approached by treating the risk factors and only at later / higher stages by surgical therapy. In case of other ischemic vascular changes (aneurysm, carotid stenosis, acute vascular occlusion), endovascular or open surgical procedures are predominating.
These types of vascular changes are amenable to invasive therapy whereas changes due to a disease are mostly treated with medication.

**Conclusion:**
Unlike other areas of pain management, many patients with vascular disease do not necessarily require prolonged therapy with analgesics.
Pain therapy is based on pain quality, previous pain intensity but rather not on age or gender.

**Pedal Bypass in Chronic Limb-Threatening Ischaemia: A Limb-Salvage Option in the Endovascular Era**
ID: E-DCK-490

**Gefäßchirurgie / Vascular Surgery**
**Gefäßchirurgie im Spannungsfeld der Krankenhausplanung / Vascular Surgery in the Context of Hospital Resource Planning**

Abhay Setia[1], Chiranthie Muhlemann[1], Samir Khan[1], Farzin Adili[1]

[1]Klinik für Gefäßmedizin, Gefäßchirurgie und Endovaskuläre Chirurgie, Klinikum Darmstadt GmbH, Klinik für Gefäßmedizin, Gefäßchirurgie und Endovaskuläre Chirurgie, Darmstadt, Deutschland

**Background:**
In the endovascular era, pedal bypass surgery is increasingly reserved for highly selected cases of chronic limb-threatening ischaemia (CLTI). We report perioperative results, patency, and wound healing outcomes following pedal bypass in this context.

**Material and methods:**
Between January 2024 and October 2025, five pedal bypass procedures were performed in patients with CLTI (mean age 76.6 years; range 72–83). Four presented with tissue loss (Fontaine IV), one with rest pain. All had diabetes mellitus; additional comorbidities included obesity (n = 3), atrial fibrillation (n = 2), coronary artery disease (n = 1), and hypercholesterolemia (n = 2).
Preoperative assessment comprised ABI, light-reflex plethysmography, duplex sonography, echocardiography, and digital subtraction angiography. Failed or early reoccluded tibial endovascular recanalization preceded surgery in two cases. In three patients, balloon angioplasty of the superficial femoral and fibular arteries was performed to optimize inflow, yet $tcPO_2$ remained low (4–26 mmHg).

**Results:**
**Operative details:**
Inflow sites were the distal SFA (n = 2), popliteal artery P3 (n = 2), and anterior tibial artery (n = 1); outflow targets were the dorsalis pedis (n = 4) and posterior tibial artery (n = 1). The reversed great saphenous vein was used as conduit in all cases (ipsilateral = 4, contralateral = 1). Mean operative time was 270 min (range 210–327), blood loss 720 mL (300–1000). Concomitant wound revision or minor amputation occurred in three cases.
**Results:**
Three patients required short-term ICU care (mean = 2 days). Early complications comprised one hematoma requiring evacuation. Mean hospital stay was 29 days (14–43). Postoperative therapy included aspirin (100 mg) plus oral anticoagulation (n = 4) or dual antiplatelet therapy (n = 1).
During follow-up (range 1–19 months), one patient died from myocardial infarction at two months. Complete wound healing occurred in one patient by 3 months and partial healing in three others (in-terms of surface wound surface area; 80% healed surface at 6 months; 20% at 1 month). Duplex ultrasonography confirmed patent grafts with high-amplitude monophasic flow in all surviving patients.
Bei so niedrigen Fallzahlen nicht %-Werte, sondern Absolutzahlen

**Conclusion:**
Despite low numbers, pedal bypass using autologous vein remains a viable limb-salvage strategy for patients with advanced CLTI when endovascular options fail. Acceptable short- to mid-term patency and wound healing can be achieved despite high operative risk and comorbidity burden.

DE GRUYTER BRILL

**Studies on the expression of fatty acid receptors 2 and 3 (FFAR2/3) in the human aorta**
ID: E-DCK-145

**Gefäßchirurgie / Vascular Surgery**
**Labor trifft Klinik / Bridging Laboratory Research and Clinical Practice**

Sophia Kollien[1], Christian Faist[2], Anton Kuhn[1], Pavel Goglev[1], Heiko Hayen[2], Alexander Oberhuber[1], Thorsten Eierhoff[1]

[1]Universitätsklinikum Münster, Klinik für Vaskuläre und Endovaskuläre Chirurgie, Münster, Deutschland,
[2]Universität Münster, Institut für Anorganische und Analytische Chemie, Münster, Deutschland

**Background:**
FFAR2/3 receptors, sensing short-chain fatty acids (SCFAs) like butyrate from the intestinal microbiota, are expressed in various tissues and organs. These receptors trigger cell- and tissue-specific signaling pathways that contribute to anti-inflammatory responses. Previous studies suggest FFAR2/3 may influence aortic endothelial integrity through VE-cadherin, potentially affecting aortic aneurysm progression. However, their expression and role in the aorta remain poorly understood.
This study aims to investigate FFAR2/3 expression in both pathological and non-pathological aortas, alongside quantifying circulating SCFAs, to determine their influence on receptor expression.

**Material and methods:**
Aortic tissue samples from patients with aortic aneurysms and from individuals without aortic pathologies were investigated. FFAR2/3 expression was analyzed by RT-qPCR. Immunohistochemical stainings and microscopic assessments of CD31 and VE-cadherin in pathologic and non-pathologic aorta were performed to validate FFAR2/3 expression along with endothelial marker proteins. HPLC-MS analysis was conducted to quantify serum SCFA concentrations in patients with and without aortic pathologies. Finally, investigations of clinical specimen were complemented by quantification of FFAR2/3 expression in human aortic endothelial cells cultured in the presence of various concentrations of butyrate.

**Results:**
Preliminary data revealed significantly lower FFAR3 expression in aortic aneurysm tissue compared to non-pathological tissue, while FFAR2 expression remained similar in both groups. Endothelial markers CD31 and VE-cadherin were expressed in both pathological and non-pathological aorta. HPLC-MS analysis showed a significant reduction of the molar ratios of butyrate in aneurysm patients compared to controls, suggesting that FFAR3 expression may be influenced by butyrate. In vitro experiments further confirmed that FFAR3 expression in human aortic endothelial cells was induced by butyrate at concentrations (5 micromolar) found in patient sera, whereas FFAR2 expression required higher butyrate levels (290 micromolar).

**Conclusion:**
Preliminary findings suggest a link between FFAR3 expression and aortic aneurysm pathology, with butyrate potentially regulating FFAR3 levels. Ongoing studies aim to confirm these results and further explore the mechanisms of FFARs.

DE GRUYTER BRILL

**Endothelial barrier function and short-chain fatty acids in aortic pathologies**
ID: E-DCK-190

**Gefäßchirurgie / Vascular Surgery**
**Labor trifft Klinik / Bridging Laboratory Research and Clinical Practice**

Anton Kuhn[1], Sophia Luise Kollien[1], Christian Faist[2], Pavel Goglev[1], Heiko Hayen[2], Alexander Oberhuber[1], Thorsten Eierhoff[1]

[1]University Hospital Muenster, Department for Vascular and Endovascular Surgery, Muenster, Deutschland, [2]University Muenster, Institute of Inorganic and Analytical Chemistry, Muenster, Deutschland

**Background:**
The regulation of endothelial barrier function is critical for vascular homeostasis and contributes to the pathogenesis of aortic aneurysm and aortic dissection. Phosphorylation of VE-cadherin and its interaction with the protein tyrosine phosphatase VE-PTP modulate endothelial permeability to solutes and immune cells. Previous studies suggest that gut microbial metabolites, such as short-chain fatty acids (SCFAs), influence the endothelial barrier. However, the underlying molecular mechanisms remain poorly understood. In vitro, we identified an SCFA-sensitive, VE-cadherin-dependent signaling pathway in human aortic endothelial cells, which affects endothelial integrity and may contribute to the pathophysiology of aortic diseases. The relationship between circulating SCFAs and endothelial barrier function remains poorly understood. This study aims to evaluate endothelial barrier status and SCFA levels in patients with and without aortic pathologies.

**Material and methods:**
Histopathological analysis was conducted to detect phosphorylated VE-cadherin, VE-PTP, CD45+ cells, and serum albumin in non-pathologic and pathological human aortic tissue samples from patients with aortic dissection or aneurysm. Fluorescence microscopy was used to quantify phospho-VE-cadherin and to examine co-localization between VE-cadherin and VE-PTP. Additionally, HPLC-MS analysis was performed to quantify serum SCFA concentrations in patients with and without aortic pathologies.

**Results:**
Preliminary data indicate an increased presence of CD45+ cells in the subintimal layer and elevated intramural serum albumin levels in aortic dissection and aneurysm samples compared to non-pathologic aortic tissue. These findings were accompanied by higher expression of phosphorylated VE-cadherin and reduced co-localization of VE-PTP with VE-cadherin in pathological samples, suggesting impaired endothelial barrier function. Moreover, CD45+ cell abundance correlated inversely with circulating butyrate concentrations.

**Conclusion:**
The results suggest a possible association between circulating SCFA levels and the integrity of endothelial cells in aortic pathologies. However, the mechanisms underlying this relationship are not yet fully understood and further research is ongoing to clarify these processes.
